# Activation of a Locus Coeruleus to Dorsal Hippocampus Noradrenergic Circuit Facilitates Associative Learning

**DOI:** 10.3389/fncel.2022.887679

**Published:** 2022-04-15

**Authors:** Theodoros Tsetsenis, Julia K. Badyna, Rebecca Li, John A. Dani

**Affiliations:** ^1^Department of Neuroscience, Mahoney Institute for Neurosciences, Perelman School for Medicine, University of Pennsylvania, Philadelphia, PA, United States; ^2^Department of Biology, School of Arts and Sciences, University of Pennsylvania, Philadelphia, PA, United States

**Keywords:** fear conditioning, optogenetics, norepinephrine, locus coeruleus, learning

## Abstract

Processing of contextual information during a new episodic event is crucial for learning and memory. Neuromodulation in the hippocampus and prefrontal cortex plays an important role in the formation of associations between environmental cues and an aversive experience. Noradrenergic neurons in the locus coeruleus send dense projections to both regions, but their contribution to contextual associative learning has not been established. Here, we utilize selective optogenetic and pharmacological manipulations to control noradrenergic transmission in the hippocampus during the encoding of a contextual fear memory. We find that boosting noradrenergic terminal release in the dorsal CA1 enhances the acquisition of contextual associative learning and that this effect requires local activation of β-adrenenergic receptors. Moreover, we show that increasing norepinephrine release can ameliorate contextual fear learning impairments caused by dopaminergic dysregulation in the hippocampus. Our data suggest that increasing of hippocampal noradrenergic activity can have important implications in the treatment of cognitive disorders that involve problems in contextual processing.

## Introduction

The contextual characteristics of an event are critical for learning and memory. Acquired information is intertwined with the learning context, which can serve as a strong retrieval cue (Smith and Bulkin, 2014). Pavlovian fear conditioning is a model of associative learning that is exhibited in both humans and animals. The hippocampus plays an essential role in the integration and processing of spatial information that is important for contextual fear conditioning (cFC) and associative learning (Kim and Fanselow, 1992). The circuits that mediate contextual associative learning in the hippocampus are subject to neuromodulation, which involves regulation of local neuronal excitability as well as synaptic plasticity (Bazzari and Parri, 2019; Likhtik and Johansen, 2019). However, very little information exists regarding the exact neuromodulatory mechanisms involved in regulating contextual associative learning in the hippocampus.

It has been proposed that catecholaminergic modulation by dopamine (DA) and norepinephrine (NE) is important for hippocampus-dependent associative learning (Thomas, 2015). Indeed, DA projections from the midbrain have been demonstrated to modulate synaptic activity in hippocampal CA1 (Rosen et al., 2015) and promote spatial memory retention (McNamara et al., 2014). Furthermore, it has been shown that dopaminergic transmission is necessary for aversive learning and associated synaptic plasticity in the hippocampus, and inhibition of D1/D5 receptors in the dorsal CA1 (dCA1) impairs performance in a passive avoidance task (Rossato et al., 2009; Broussard et al., 2016). Recent evidence indicates that DA originating from the midbrain regulates the acquisition of contextual fear learning in the dorsal hippocampus (Tsetsenis et al., 2021). On the other hand, NE has also been implicated in the modulation of hippocampus-dependent memory processes (Lemon et al., 2009; Sara, 2009). In the brain, the majority of NE is synthesized and released by the locus coeruleus (LC), a small bilateral nucleus located in the brainstem. The LC also produces DA as a precursor of norepinephrine (NE), and can corelease both neurotransmitters in the hippocampus, acting as an alternative source of DA (Smith and Greene, 2012; Kempadoo et al., 2016; Takeuchi et al., 2016). In fact, it was shown that optogenetic activation of tyrosine hydroxylase (TH) positive neurons in the LC enhances hippocampal spatial memory (Kempadoo et al., 2016; Takeuchi et al., 2016) and promotes contextual aversive generalization in the dentate gyrus (Seo et al., 2021). However, genetic ablation of catecholamine production exclusively in LC-NE neurons has no effect in the encoding of contextual fear memory, indicating that NE activity is dispensable for associative learning in the hippocampus (Tsetsenis et al., 2021). Despite this evidence, the effect of specific activation of LC-NE activity on hippocampus-dependent contextual associative learning has not been directly tested.

Here, we utilize viral tracing methodology to identify TH-positive neurons anatomically in the LC that send axonal projections to the dorsal hippocampus. Optogenetic activation of neurotransmitter release from the terminals of these neurons during the encoding phase of cFC enhances associative fear memory. Combining optogenetics with pharmacology, we demonstrate that this enhancement is mediated by local hippocampal beta-adrenergic signaling. Our data indicate that NE release in the dorsal hippocampus has the ability to facilitate contextual fear memory formation and can reverse the impairment caused by inhibition of dopaminergic signaling in this area.

## Materials and Methods

### Mice

C57BL/6J (RRID:IMSR_JAX:000664) and Ai14 (RRID:IMSR_JAX:007914) mice were purchased from the Jackson Laboratory. Dbh-Cre transgenic mice were obtained from the Mutant Mouse Regional Resource Center (RRID:MMRRC_036778-UCD). Adult male mice (8–16 weeks old) were used for behavioral experiments and both male and female mice (8–12 weeks old) were used for anatomical tracing. Mice were housed on a 12-h light/dark cycle (lights on at 21:00) with food and water available *ad libitum*. All behavioral procedures were performed during the animals’ dark cycle. Mice were allowed to acclimate to the test room for at least 30 min before initiating behavioral procedures. All experiments complied with the animal care standards of the National Institutes of Health and were approved by the Institutional Animal Care and Use Committee (IACUC) of the University.

### Stereotactic Surgeries

All stereotactic procedures were performed under inhaled isoflurane anesthesia and using a stereotaxic instrument (Angle Two, Leica Biosystems, Deer Park, IL, United States). Body temperature was kept stable by using a feedback-controlled heating pad during surgery and while recovering from anesthesia. For optogenetics experiments, 0.5–1 μL of adeno-associated virus (AAV) solution [AAV5-EF1a–DIO-hChR2(H134R)-eYFP; AAV5-EF1a-DIO-eYFP; approximately 10^12^ infectious units per mL, prepared by the University of North Carolina Vector Core Facility] was injected bilaterally into the locus coeruleus (LC; lambda: −0.80 mm, lateral: ±0.80 mm, ventral: −3.60 mm) at a rate of 0.2 μL/min using a syringe pump (KD Scientific, Holliston, MA, United States). After the end of the infusion, the needle was left at the injection site for an additional 10 min before being withdrawn. Following the completion of viral injections, mice received bilateral implantation of optical fibers (200 μm diameter; NA = 0.50; Thorlabs, Newton, NJ, United States) above the dorsal part of hippocampal CA1 (dCA1; bregma: −1.45 mm, lateral: ±1.00 mm, ventral: −1.05 mm, angle: ±5°). For combined optogenetic and pharmacological experiments, a bilateral guide cannula (26 gauge, 0.48 mm outer diameter, 0.32 mm inner diameter, 3 mm long; 2 mm center to center; Plastics One, Roanoke, VA, United States) was implanted in the same region. Optic fibers and cannulas were secured to the scull with adhesive cement (C&B Metabond, Parkell, Edgewood, NY, United States). For retrograde tracing and labeling, 1 μL of canine associated virus (CAV) solution (CAV2-Cre; CAV2-Flex-Flpo; approximately 5 × 10^12^ physical particles per mL, prepared by the Vector Platform of the University of Montpellier) was injected into the dorsal hippocampus (dHip; bregma: −1.80 mm, lateral: 1.00 mm, ventral: −1.50 mm). Experiments were performed 8–12 weeks (for AAVs) or 3–5 days (for CAV-2) after stereotactic injection. Coronal sections (50–70 μm in thickness) from all injected and implanted mice were histologically examined to verify proper injection, optical fiber, and cannula placements.

### Fear Conditioning

Contextual fear conditioning was performed in a soundproof, red-lit room during the dark cycle of the mice. The mice were acclimated to the room for thirty minutes before fear conditioning training and testing. Before each training session the fear conditioning chamber was cleaned with 20% ethanol solution. The grid floor of the chamber was connected to a shock generator (Coulbourn, Holliston, MA, United States). The training protocol allowed the mouse two minutes to become acclimated to the chamber followed by a nine-minute period over which five foot shocks (0.4 mA, 1 s) were delivered in pseudorandomized intervals averaging 2 min. After the delivery of the last foot shock, mice remained in the chamber for an additional minute before returning to their home cage. For optogenetic manipulations, blue light from a 450 nm laser (CNI, Changchun, China) was delivered at 10 mW power (measured at the tip of the optic fibers) to stimulate neuronal fibers in the dCA1. Light pulses were delivered *via* bilateral optic fiber cables (Doric Lenses, Quebec, Canada) coupled to the optic fiber cannulas that were implanted to each mouse. A pulse generator connected to the laser was activated by a TTL signal originating from the fear conditioning software (FreezeFrame, Actimetrics, Wilmette, IL, United States). This TTL signal was delivered together with the first foot shock and triggered blue light pulses (10 ms at 20 Hz for 1 s every 10 s) that continued until the end of the training protocol. All mice received light stimulation during training. Testing occurred approximately 24 h after training, with all the mice returning to the same fear-conditioning chamber for a total period of 5 min. Mice were again connected with optic fiber cables but this time no light was delivered. Fear conditioning testing in a different context occurred 24 h later. Mice were placed for 2 min in a different chamber (floor and walls were changed and vanilla scent was sprayed in the chamber) with no optic fiber cables attached. For every session, the mouse behavior was recorded, and the amount of time the mouse spent freezing (immobility bouts greater than 1 s) was calculated using the FreezeFrame software. Freezing was scored by an individual who was unaware of the experimental conditions, animal genotypes, or treatments.

### Local Drug Infusions and Optopharmacology

All drug infusions and optopharmacology behavioral tasks were performed in a sound-vaulted, red-lit room during the dark cycle for the mice as described previously (Broussard et al., 2016). The mice were acclimated to the room for thirty minutes before training and testing. Depending on the experiment, mice received bilateral infusion of either sterile saline, SCH23390 (1 mg/mL), or propranolol (6.25 mg/mL) into the dCA1. Infusions were performed at a rate of 0.25 μL/min (total volume of 0.5 μL per side) with a syringe pump (Harvard Apparatus, Holliston, MA, United States) in the animal’s home cage. The injector was left inserted for 5 mins following completion of the infusion to allow for drug diffusion. The mouse was then allowed to stay in its home cage for an additional fifteen minutes. During this time, the injector was tested to ensure that proper delivery had occurred by running a test infusion through the internal cannula. After the 15-min incubation, mice were subjected to cFC training following the same fear conditioning protocol described above. For each mouse, optic fiber wires coupled with an optogenetics housing cap (Plastics One, Roanoke, VA, United States) were inserted above the dCA1 through the same guide cannula used for the drug infusions. All mice received light stimulation during training.

### Histology, Immunofluorescence, and Imaging

Histological processing, immunofluorescence staining, and microscopy were performed as described previously (Tsetsenis et al., 2011). Mice were perfused intracardially with 4% paraformaldehyde in PBS, pH 7.4, and their brains were removed and post-fixed overnight. Coronal sections (50–70 mm) from hippocampus and LC were prepared in a vibratome (Leica Biosystems, Deer Park, IL, United States). Sections were stained overnight with primary antibodies against GFP (mouse anti-GFP, 1:1,000, Thermo Fisher Scientific, Waltham, MA, United States, A11120) and tyrosine hydroxylase (rabbit anti-TH, 1:500, Millipore, Burlington, MA, United States, AB152). The next day, sections were incubated with compatible Alexa Fluor goat secondary antibodies (1:250, Thermo Fisher Scientific, Waltham, MA, United States) for 2 h. For high resolution images, acquisition was performed with a Leica SP5 laser scanning confocal microscope using 40× or 60× objectives. For lower resolution, images were taken using 5× and 10× objectives of an Olympus BX63 automated fluorescence microscope. All images were processed with ImageJ and sections were labeled relative to bregma according to “The Mouse Brain in Stereotaxic Coordinates” (Franklin and Paxinos, 2008). Maximum z-projections of confocal image stacks approximately 30 μm in thickness with overlaying color channels of interest were used to manually count neuronal cell bodies and determine colocalization.

### Statistical Analysis

Statistical analyses were performed with Prism (Graphpad, San Diego, CA, United States). For comparisons between two groups, two-tailed unpaired *t* tests were used, whereas differences across more than two groups were analyzed with analysis of variance (ANOVA) followed by *post hoc* tests in case of significance to account for multiple comparisons. All experimental data are reported as means ± SEM. Differences where *p* < 0.05 are considered statistically significant.

## Results

### Tyrosine Hydroxylase-Positive Locus Coeruleus Neurons Send Axonal Projections to the Dorsal Hippocampus

**FIGURE 1:**
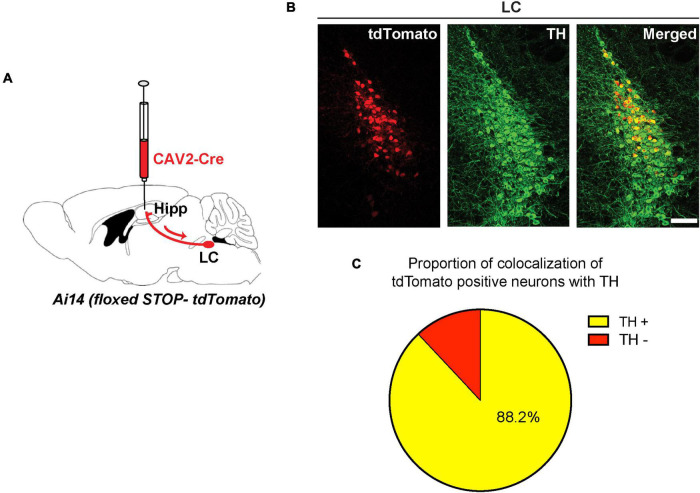
Retrogade viral tracing identifies locus coeruleus (LC) TH-positive neurons projecting to the dorsal hippocampus. **(A)** CAV2-Cre injected in the hippocampus of Ai14 reporter mice to allow retrograde labeling of projection neurons. **(B)** Coronal sections from the LC showing retrogradely labeled cells (tdTomato-positive, red) were counterstained with an anti-TH antibody (green); scale bar, 100 μm. **(C)** Percentage of tdTomato-positive LC neurons that are also positive for TH (yellow, tdTomato/TH double-positive: 88.2 ± 1.1%, *n* = 3 mice).

To identify LC neurons that send direct projections into the dorsal hippocampus, we utilized a viral retrograde labeling approach (Soudais et al., 2001). We injected a canine adenovirus type 2 expressing Cre recombinase (CAV2-Cre) in the dorsal hippocampus of Ai14 reporter mice (Madisen et al., 2010; Figure 1A). In these mice, retrograde transfer of Cre-expressing viral particles activates the expression of tdTomato in hippocampus-projecting neurons. We then analyzed sections from the LC of these mice after immunostaining with an antibody against TH (Figure 1B). Using manual counting and a custom-written ImageJ script, we found a high degree of co-localization of TH with tdTomato (Figure 1C). These data suggest that a large population of the retrogradely labeled tdTomato-positive neurons in the LC (i.e., 88.2%) were TH-positive neurons that send projections into the dorsal hippocampus.

**FIGURE 2:**
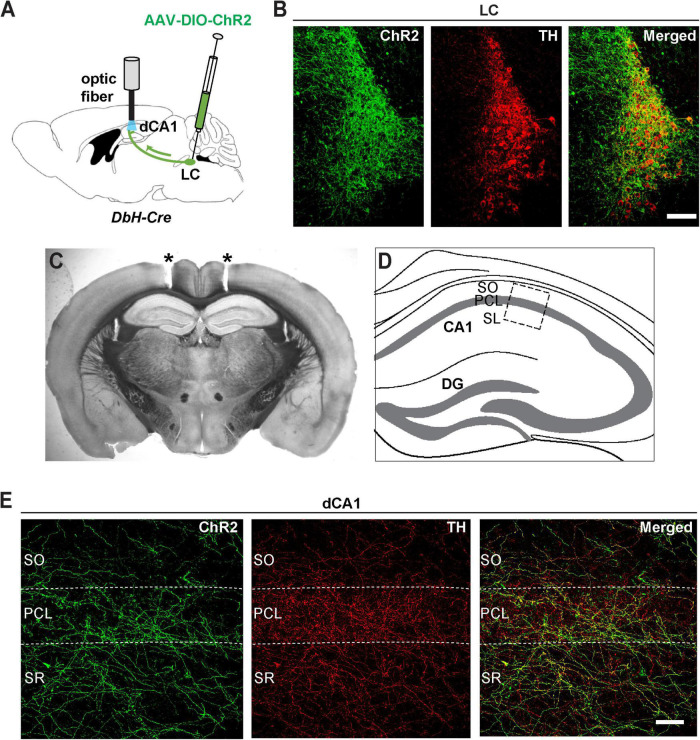
Viral expression of ChR2-eYFP shows noradrenergic innervation of dorsal CA1 from LC. **(A)** An AAV encoding Cre-dependent ChR2-eYFP was bilaterally injected in the LC of Dbh-Cre mice and optic fibers were implanted above the dCA1. **(B)** Coronal sections from the LC of a Dbh-Cre mouse injected with AAV-DIO-ChR2-eYFP were immunostained with antibodies against GFP (to enhance eYFP fluorescence of ChR2 expression, green) and TH (red); scale bar, 100 μm. **(C)** Representative coronal section of a Dbh-Cre mouse showing tracts (asterisks) of bilateral optic fiber placements above the dCA1 field. **(D)** Schematic illustration of the dorsal hippocampus with a boxed area that corresponds to the dCA1 region of the high-magnification images shown in panel **(E)**. **(E)** Confocal images of the dCA1 from the same mouse as in B showing expression of ChR2 positive fibers (green) that co-localize with TH (red); SO, stratum oriens, PCL, pyramidal cell layer, SR, stratum radiatum; scale bar, 50 μm.

To visualize these noradrenergic axonal projections, we injected channelrhodopsin (AAV-DIO-ChR2-YFP) into the LC of dopamine beta-hydroxylase (Dbh-Cre) mice (Figure 2A). In this case, Cre-dependent ChR2 expression is restricted to LC noradrenergic neurons that express the NE biosynthetic enzyme Dbh (Schwarz et al., 2015). Thus, ChR2 expression in the LC of these mice (ChR2*^LC^*) was restricted to cells co-expressing TH (Figure 2B). Consistent with our retrograde tracing analysis (Figure 1), we found a dense network of axonal fibers in the dCA1 that co-expressed ChR2 and TH (Figures 2D,E), indicating that LC neurons send axonal projections to this region.

### Optogenetic Activation of Locus Coeruleus Terminals in the Dorsal CA1 Enhances Acquisition of Contextual Fear Memory

**FIGURE 3:**
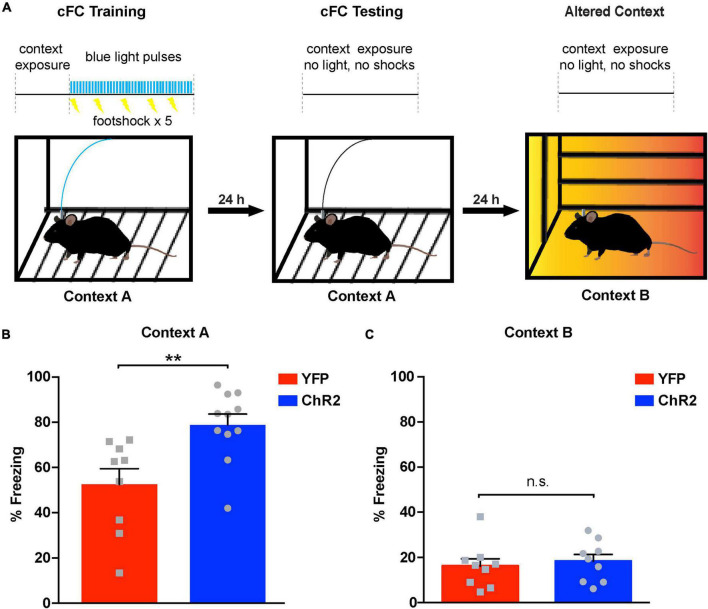
Optogenetic activation of noradrenergic terminals in dCA1 during acquisition of cFC enhances freezing 24 h later. **(A)** Schematic illustration of cFC procedure. A horizontal black line with light trains as vertical blue bars and shocks as lightning bolts represents the timeline of the exposure to each context. Blue light delivery and shocking occurred only during training. **(B)** Bar graph showing freezing responses of ChR2*^LC^* and YFP*^LC^* (control) mice after re-exposure to the aversive context “A” 24 h after training. Unpaired Student’s *t*-test, *p* = 0.003; *n* = 12, 11 for YFP and ChR2 respectively; **p < 0.01; data represent means ± SEM. **(C)** Graph showing freezing responses of ChR2*^LC^* and YFP*^LC^* mice in an altered context “B” 24 h after re-exposure to context “A”. Unpaired Student’s *t*-test, *p* = 0.634; *n* = 9 for YFP and ChR2; n.s.: *p* > 0.05; data represent means ± SEM.

Contextual fear conditioning (cFC) is a behavioral paradigm that can be used to assess associative learning in rodents and humans (Fanselow and Poulos, 2005). It has been shown that cFC depends strongly on hippocampal function (Kim and Fanselow, 1992; Maren et al., 1997), especially on dCA1 (Goshen et al., 2011) where it can induce synaptic plasticity in the form of long-term potentiation (Subramaniyan et al., 2021). Therefore, as a next step, we asked whether augmenting release from LC terminals in the dCA1 has an effect on cFC acquisition and associative memory formation. To this end, we implanted optic fiber cannulas above the dCA1 of ChR2*^LC^* and YFP-injected (YFP*^LC^*) control mice (Figures 2A,C) and trained them using a specialized cFC protocol (Figure 3A). When we measured freezing responses in these mice 24 h after training, we found that ChR2*^LC^* mice exhibited significant higher levels of freezing compared to controls (blue bar, Figure 3B; Supplementary Movie 1). We then examined the possibility that this effect is context-independent by introducing the mice in a modified chamber (Context B) 24 h after testing (Figure 3A). None of the groups exhibited substantial freezing (Figure 3C), excluding the possibility that our manipulation causes generalized fear responses. Thus, increased release from LC terminals in the dCA1 modulates cFC acquisition and strengthens the association between contextual cues and the aversive experience, facilitating associative learning.

### Noradrenergic Stimulation in the Dorsal CA1 Facilitates Contextual Fear Learning *via* Activation of Beta Adrenergic Receptors

Our data suggest that stimulating release from LC terminals in dCA1 during cFC training results in enhanced fear responses during recall, in a mode similar to what was previously observed for midbrain DA (Tsetsenis et al., 2021). Although NE is the principal neurotransmitter released from LC terminals, evidence indicates that DA is also released from LC terminals in the hippocampus (Kempadoo et al., 2016; Takeuchi et al., 2016). Thus, we could not exclude the possibility that DA co-release from LC terminals mediates the enhanced cFC memory observed in Figure 3B. To test this possibility, we devised a strategy combining optogenetics with pharmacology to dissociate the effects of the two different neuromodulators being released due to our optogenetic manipulations of LC terminals.

**FIGURE 4:**
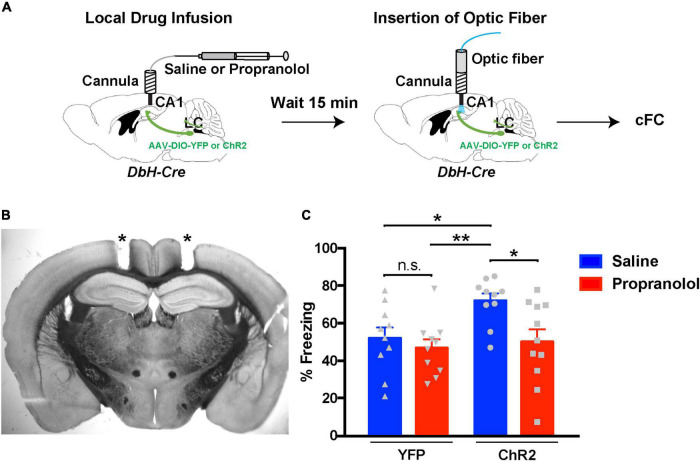
Inhibition of hippocampal β-adrenergic receptors reverses the effects of optogenetic activation of LC terminals. **(A)** Illustration of the procedure for drug infusions and contextual fear conditioning with optogenetics. **(B)** Representative coronal section of a Dbh-Cre mouse showing tracts (asterisks) of the bilateral cannula placement above the dCA1 field. **(C)** Graph showing freezing responses of ChR2*^LC^* and YFP*^LC^* (control) mice infused with saline (vehicle) or propranolol 24 h after treatment and cFC training. All mice received blue-light activation during training. Two-Way ANOVA for drug treatment: *F*_1_,_37_ = 6.248; *p* = 0.017; Dunnet’s multiple comparisons test: ChR2-saline vs ChR2-propranolol, *p* = 0.018; ChR2-saline vs YFP-saline, *p* = 0.041; ChR2-saline vs YFP-propranolol, *p* = 0.007; *n* = 10 for ChR2-saline, YFP-saline and YFP-propranolol, *n* = 11 for ChR2-propranolol; *p < 0.05, **p < 0.01; n.s.: *p* > 0.05; data represent means ± SEM.

Our aim was to stimulate release optogenetically from LC terminals in dCA1 during cFC acquisition while inhibiting either dopaminergic or noradrenergic signaling in the same area. A conundrum that arises with this strategy is that D1/D5 receptor inhibition is causing an impairment in cFC (Heath et al., 2015; Tsetsenis et al., 2021), and that impairment could potentially confound our results. Therefore, we first tested the hypothesis that the facilitation of cFC memory due to optogenetic activation of LC terminals is mediated *via* NE signaling. We injected Dbh-Cre mice in the LC with AAVs expressing Cre-dependent versions of ChR2 or YFP (as control) and implanted bilateral cannulas over the dCA1 (Figures 4A,B). These mice received local infusion of propranolol (to inhibit β-adrenergic receptors) or saline (control) 15 min prior to cFC training. Using the same bilateral cannulas as guides, we inserted optic fibers above the dCA1 and started cFC training following the same protocol as for the previous optogenetic procedures (Figure 3A). When we measured freezing responses 24 h later, our data confirmed our previous observation (Figure 3B) that activation of release from LC terminals in the dCA1 during training results in enhanced fear memory recall (Figure 4C, ChR2 blue bar). However, this effect is abolished when we block beta-adrenergic receptors in dCA1 (Figure 4C, ChR2 red bar), suggesting that this phenotype is mediated by NE signaling through these beta-adrenergic receptors. In the control case, with YFP expressed in LC neurons, the cFC-induced freezing was the same with or without inhibition of the β-adrenergic receptors by propranolol (Figure 4C, YFP red bar). These data demonstrate that increased NE release from LC terminals in dCA1 is sufficient to enhance contextual fear memory formation (Figure 4C, ChR2), but unlike D1/D5 inhibition, the β-adrenergic signal had no measurable effect under baseline conditions (Figure 4C, YFP).

### Noradrenergic Stimulation in the Dorsal CA1 Rescues Contextual Learning Impairments Caused by Inhibition of Dopaminergic Signaling

**FIGURE 5:**
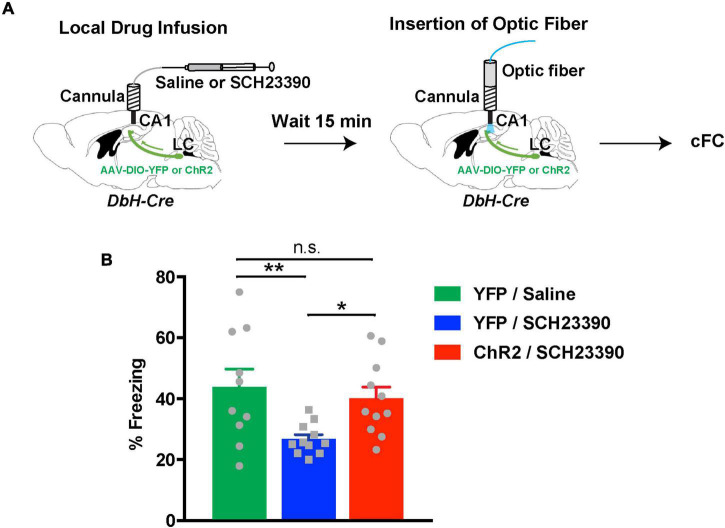
Noradrenergic stimulation rescues contextual learning impairments caused by D1/D5 receptor inhibition in the hippocampus. **(A)** Illustration of the procedure for drug infusions and cFC with optogenetics. **(B)** Graph showing freezing responses of ChR2*^LC^* and YFP*^LC^* mice infused with D1/D5 receptors inhibited by SCH23390 24 h after treatment and cFC training. All mice received blue-light activation during training. The shaded green horizontal bar represents freezing of saline-treated YFP*^LC^* control mice (mean ± SEM from Figure 5B). One-Way ANOVA for treatment: *F*_2_,_29_ = 5.001; *p* = 0.014; Fisher’s LSD: YFP/saline vs YFP/SCH23390, *p* = 0.006; ChR2/SCH23390 vs YFP/SCH23390, *p* = 0.022; ChR2/SCH23390 vs YFP/saline, *p* = 0.562; *n* = 10 for YFP/saline, *n* = 11 for YFP/SCH23390 and ChR2/SCH23390; *p < 0.05, **p < 0.01; n.s.: *p* > 0.05; data represent means ± SEM.

The above results were surprising and extend previous studies, which support that DA but not NE release in dCA1 from LC terminals facilitates spatial memory retention (Kempadoo et al., 2016; Takeuchi et al., 2016). To verify our finding, we asked whether NE release was sufficient to facilitate contextual fear memory formation in the absence of dopaminergic signaling. As we previously showed (Tsetsenis et al., 2021), inhibition of D1/D5 receptors in dCA1 during cFC training causes an amnesic effect during recall. Therefore, we infused ChR2*^LC^* and YFP*^LC^* mice with the D1/D5 receptor antagonist SCH23390 15 min prior to cFC training (Figure 5A). During cFC training we activated release from LC terminals with light as before. As expected, SCH23390-treated YFP*^LC^* mice showed decreased levels of freezing during testing the next day because local dopaminergic signaling had been inhibited (Figure 5B, blue bar). On the other hand, activation of release from LC terminals in ChR2*^LC^* mice reversed the impairment in contextual fear memory formation caused by D1/D5 receptor inhibition and resulted in normal freezing during testing (Figure 5B, red bar). Taken together, our results indicate that activation of NE release can act locally in dCA1 and facilitate contextual associative learning in cFC. Moreover, optogenetic stimulation of NE release substantially ameliorates the impairment in contextual fear memory formation caused by inhibition of dopaminergic signaling in dCA1.

## Discussion

Although midbrain dopaminergic centers were long thought to be the origin of hippocampal DA, recent studies indicate the existence of a second source (Kempadoo et al., 2016; Takeuchi et al., 2016). Those studies showed that noradrenergic fibers from the LC can co-release DA in the hippocampus, and this phenomenon can modulate spatial memory retention. Specifically, optogenetic activation of the LC or LC-TH^+^ hippocampal fibers enhanced spatial learning in a D1/D5 receptor dependent and β-adrenergic independent manner. On the other hand, it was also shown that optogenetic activation of VTA-TH^+^ terminals in the dorsal hippocampus can also enhance special memory retention in a different task (McNamara et al., 2014). Collectively, these data provide insights for the existence of two DA sources that act in the hippocampus *via* similar signaling mechanisms but could modulate different aspects of learning (McNamara and Dupret, 2017; Duszkiewicz et al., 2019). Moreover, the evidence indicates that NE – the main neurotransmitter released from LC-TH^+^ hippocampal terminals – is not involved in the observed enhancement of spatial memory retention (Kempadoo et al., 2016; Takeuchi et al., 2016). However, it is unclear whether the same principles exist regarding neuromodulation of aversive memory formation in the hippocampus.

In the present study, we demonstrate that optogenetic activation of neurotransmitter release from LC–TH^+^ hippocampal terminals during the encoding phase of contextual fear conditioning enhances fear responses during recall (Figure 3). We show that this enhancement can be reversed with local inhibition of β-adrenergic signaling in dCA1 by propranolol (Figure 4), indicating that this effect is mainly attributed to NE action. Furthermore, optogenetic release from LC–TH^+^ terminals in dCA1 can act in the presence of D1/D5 receptor inhibition and is sufficient to ameliorate the aversive learning impairment caused by blockade of DA signaling (Figure 5). Taken together these data provide evidence that NE originating from the LC, similar to midbrain DA, can exert neuromodulatory action during aversive memory acquisition resulting in enhanced fear responses during recall the next day. However, the issue is complicated by the finding that under baseline conditions (Figure 4C, YFP) inhibition of β-adrenergic signaling by propranolol does not significantly decrease the fear response during recall. This finding suggests that β-adrenergic signaling is serving as an alternative or safety for the normally engaged DA signaling that directly modulates the cFC memory.

Since the entire hippocampal formation receives dense innervation from the LC, we designed our optic fiber implantation to avoid activation of the CA3 and DG subregions. We implanted the optic fibers with a 5° incline away from the CA3 and approximately 0.75 mm from the upper border of the DG. At this distance only 1% of the light power remains (Aravanis et al., 2007) which is too low to induce ChR2 currents (Lin, 2011). Thus, the probability of our light stimulation activating release from LC terminals outside the CA1 is very low.

Although the idea that stimulation of NE release in the dCA1 enhances aversive memory formation is in contrast with what was shown for spatial memory retention, it should not be surprising. Activation of β-adrenergic receptors by NE has been shown to facilitate LTP in the hippocampus (Qian et al., 2012; Hansen and Manahan-Vaughan, 2015; Liu et al., 2017). NE activation of β-adrenergic receptors in the hippocampus is also required during recall of aversive memory. Systemic administration or local hippocampal infusion of propranolol before cFC testing resulted in low freezing responses compared to controls (Murchison et al., 2004). On the other hand, local or systemic inhibition of β-adrenergic signaling during the encoding phase of cFC and inhibitory avoidance had no effect on recall (Murchison et al., 2004; Broussard et al., 2016). These results are in agreement with our data (Figure 4C, YFP), indicating that in contrast with hippocampal DA, NE is not vital for the acquisition phase of aversive memory formation. Nevertheless, it has also been shown that systemic administration of NE facilitates cFC and enhances aversive memory formation in mice (Frankland et al., 2004b; Hu et al., 2007). Along those lines, our data show that stimulation of hippocampal NE release during cFC encoding is sufficient to enhance freezing responses during recall (Figure 3B). As in the case of DA (Tsetsenis et al., 2021), this effect is context-dependent (Figure 3C), precluding the possibility that this is a generalized fear effect.

How do DA and NE exert similar effects on hippocampal regulation of aversive memory formation? It is possible that DA through the activation of D1-like receptors and NE *via* the activation of β-adrenergic receptors can trigger converging signaling pathways in the dCA1. Both receptor classes are G_α*s*_-coupled and can act *via* cAMP/PKA/MEK pathways to facilitate plasticity in the hippocampus. It is known that activation of these receptors can enhance NMDAR function either *via* inhibition of potassium channels (Watanabe et al., 2002; Yuan et al., 2002; Yang and Dani, 2014) or by direct phosphorylation of NMDARs by PKA (Murphy et al., 2014). Additionally, PKA promotes the phosphorylation of AMPAR subunits (Esteban et al., 2003; Hu et al., 2007), providing another mechanism for LTP facilitation. Finally, activation of PKA induces protein synthesis (Kelleher et al., 2004; Smith et al., 2005), providing another converging pathway by which DA and NE could modulate hippocampal plasticity through an expanded time window that would explain their delayed effects on aversive memory formation.

Our data do not exclude the possibility that co-release of DA from LC terminals is also involved in the enhancement in aversive memory retention. Although co-release of DA and NE from LC terminals is likely, we examined the impact of the adrenergic contribution by blocking DA signaling while optogenetically stimulating LC hippocampal fibers during cFC acquisition (Figure 5). Optogenetic stimulation of LC terminals was able to increase the depressed freezing responses caused by D1-like inhibition. Thus, NE release from LC terminals significantly restored the initial impairment in aversive memory recall caused by D1-like inhibition, indicating that NE release is sufficient to compensate for DA’s action in dCA1. These data could potentially have important implications in pathological conditions characterized by dopaminergic dysfunction. In fact, a recent study has identified that degeneration of midbrain DA neurons contributes to memory deficits and impairment in CA1 synaptic plasticity in a mouse model of Alzheimer’s disease (Nobili et al., 2017). In this and other cases, where the LC remains unaffected, boosting NE levels in the hippocampus could ameliorate and possibly reverse some of the adverse effects of dopaminergic dysregulation.

### Future Directions Regarding Hippocampus-Prefrontal Cortex Connectivity and Episodic Memory Encoding

Coordinated activity between the Hippocampus (HPC) and Prefrontal Cortex (PFC) is important for many cognitive functions, and it is affected in several neurological and psychiatric disorders (Kovner et al., 2019). These two areas have been shown to interact bidirectionally through oscillatory synchrony, which links them during memory replay (Siapas et al., 2005). Of particular interest is the fundamental role of the HPC-PFC network in episodic memory, which strongly depends on context associations with daily experiences (McClelland et al., 1995; Eichenbaum, 2000). Initial research has provided strong evidence of a functional interaction between the HPC and PFC during memory consolidation and retrieval, supporting the transfer of memories initially stored in the hippocampus to the PFC for long-term storage and recall (Frankland et al., 2004a; Wiltgen et al., 2004). Until recently, however, the contribution of PFC to memory encoding has not received detailed attention.

Several lines of evidence underscore the importance of PFC and PFC-HPC interactions during the encoding of new episodic memories. Fear conditioning training was demonstrated to induce LTP in CA1 to PFC projections (Doyere et al., 1993), while inhibition of PFC activity during FC encoding impairs memory recall (Tang et al., 2005; Zhao et al., 2005; Einarsson and Nader, 2012; Bero et al., 2014). Brain-wide mapping of c-Fos induction after cFC encoding revealed increased activity in the PFC (Cho et al., 2017). Acquisition of cFC also induces transcriptional changes and structural plasticity in the PFC (Vetere et al., 2011; Bero et al., 2014). In line with these results, fear conditioning training induces phosphorylation of the extracellular signal-regulated kinase (ERK) in the PFC, and pharmacological inhibition of ERK activity during conditioning impairs memory formation (Runyan et al., 2004). Moreover, perturbations in the connectivity between the PFC and HPC impair the formation of different types of association recognition memory (Barker and Warburton, 2015; de Souza Silva et al., 2016) including contextual fear memories (Twining et al., 2020).

In addition to the above evidence supporting a functional interplay between HPC and PFC during associative learning, neuromodulation of the HPC-PFC pathway seems to play an essential role to the fine-tuning of the circuit, and its dysregulation is implicated in neuropsychiatric disorders (Godsil et al., 2013; Ruggiero et al., 2021). Among other neuromodulators, the PFC, like the HPC, receives strong NE innervation, which has been shown to influence cognitive functions (Chamberlain and Robbins, 2013; Nguyen and Connor, 2019). However, not much is known about the role of NE in the PFC and the PFC-HPC pathway regarding the modulation of associative learning and contextual fear memory encoding. Based on the data we present here, future endeavors should aim to investigate whether and how PFC contributes to the facilitation of associative learning we observed when activating NE release in the dorsal CA1. It is possible that these effects are mediated by HPC-PFC interactions through activity and other changes in this circuit.

## Data Availability Statement

The original contributions presented in the study are included in the article/Supplementary Material, further inquiries can be directed to the corresponding authors.

## Ethics Statement

The animal study was reviewed and approved by Institutional Animal Care and Use Committee (IACUC) of the University of Pennsylvania.

## Author Contributions

TT and JD designed the research. TT, JB, and RL performed the research. TT analyzed the data and wrote the manuscript with feedback from JD. All authors contributed to the article and approved the submitted version.

## Conflict of Interest

The authors declare that the research was conducted in the absence of any commercial or financial relationships that could be construed as a potential conflict of interest.

## Publisher’s Note

All claims expressed in this article are solely those of the authors and do not necessarily represent those of their affiliated organizations, or those of the publisher, the editors and the reviewers. Any product that may be evaluated in this article, or claim that may be made by its manufacturer, is not guaranteed or endorsed by the publisher.

## Supplementary Material

Supplementary Movie 1 Representative examples showing freezing responses of ChR2*^LC^* and YFP*^LC^* mice during the first minute of re-exposure to the shocking chamber.
